# *QuickStats:* Age-Adjusted Percentage* of Adults Aged ≥18 Years Who Reported That They Needed Dental Care During the Past 12 Months But Didn’t Get It Because They Couldn’t Afford It,^†^ by Sex, Race, and Hispanic Origin^§^ — National Health Interview Survey, 2017^¶^

**DOI:** 10.15585/mmwr.mm6811a4

**Published:** 2019-03-22

**Figure:**
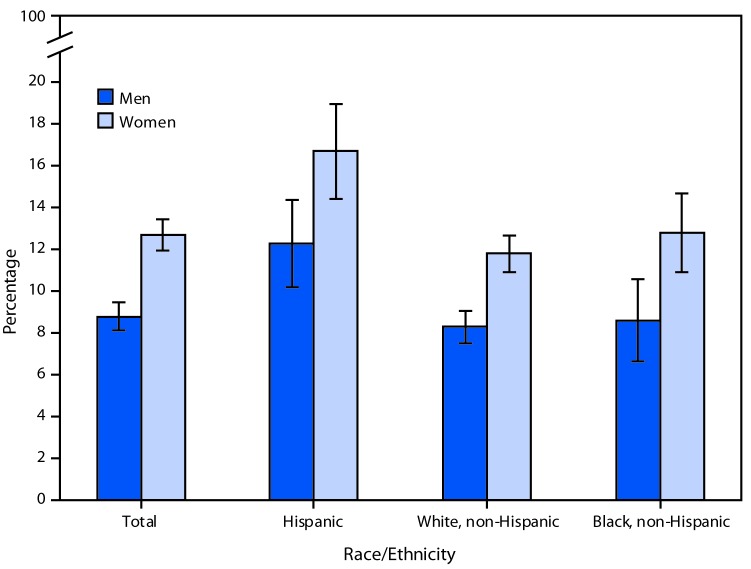
In 2017, more women (12.7%) than men (8.8%) reported that at some time during the past 12 months they needed dental care but didn’t get it because they couldn’t afford it. This pattern was consistent within each racial/ethnic group: Hispanic, non-Hispanic white, and non-Hispanic black. Among both men and women, Hispanic adults were most likely to have unmet needs for dental care because they couldn’t afford it. Nearly 17% of Hispanic women could not afford to meet their dental care needs, compared with 12.8% of non-Hispanic black women and 11.8% of non-Hispanic white women; 12.3% of Hispanic men had unmet dental care needs, compared with 8.6% of non-Hispanic black men and 8.3% of non-Hispanic white men.

**Source:** Tables of Summary Health Statistics, 2017. https://ftp.cdc.gov/pub/Health_Statistics/NCHS/NHIS/SHS/2017_SHS_Table_A-19.pdf.

